# Probing the oligomeric re-assembling of bacterial fimbriae in vitro: a small-angle X-ray scattering and analytical ultracentrifugation study

**DOI:** 10.1007/s00249-021-01543-3

**Published:** 2021-05-04

**Authors:** Alexandra S. Solovyova, Daniel T. Peters, Gema Dura, Helen Waller, Jeremy H. Lakey, David A. Fulton

**Affiliations:** 1 Proteome and Protein Analysis, Medical School, Newcastle University, Framlington Place, Newcastle-upon-Tyne, NE2 4HH UK; 2 Biosciences Institute, Medical School, Newcastle University, Framlington Place, Newcastle-upon-Tyne, NE2 4HH UK; 3 Chemistry, School of Natural and Environmental Sciences, Newcastle University, Newcastle-upon-Tyne, NE1 7RU UK; 4 Present Address: Departamento de Química Inorgánica, Orgánica y Bioquímica, Universidad de Castilla-La Mancha, Facultad de Ciencias yTecnologías Químicas-IRICA, Avda. C. J. Cela, 10, 13071 Ciudad Real, Spain

**Keywords:** Oligomeric growth, Repolymerisation, Mass increment, Flexibility

## Abstract

Capsular antigen fragment 1 (Caf1) is an oligomeric protein consisting of 15 kDa monomeric subunits that are non-covalently linked through exceptionally strong and kinetically inert interactions into a linear polymer chain. It has been shown that after its thermal depolymerisation into unfolded monomeric subunits, Caf1 is able to efficiently repolymerise in vitro to reform its polymeric structure. However, little is known about the nature of the repolymerisation process. An improved understanding of this process will lead to the development of methods to better control the lengths of the repolymerised species, and ultimately, to better design of the properties of Caf1-based materials. Here we utilize small-angle X-ray scattering to estimate the size of Caf1 polymers during the first 24 h of the re-polymerisation process. Analytical ultracentrifugation measurements were also used to investigate the process post-24 h, where the rate of repolymerisation becomes considerably slower. Results show that in vitro polymerisation proceeds in a linear manner with no evidence observed for the formation of a lateral polymer network or uncontrolled aggregates. The rate of Caf1 in vitro repolymerisation was found to be concentration-dependent. Importantly, the rate of polymer growth was found to be relatively fast over the first few hours, before continuing at a dramatically slower rate. This observation is not consistent with the previously proposed step-growth mechanism of in vitro polymerisation of Caf1, where a linear increase in polymer length would be expected with time. We speculate how our observations may support the idea that the polymerisation process may be occurring at the ends of the chains with monomers adding sequentially. Our findings will contribute towards the development of new biomaterials for 3D cell culture and bio-printing.

**Supplementary Information:**

The online version contains supplementary material available at 10.1007/s00249-021-01543-3.

## Introduction

**Fig. 1:**
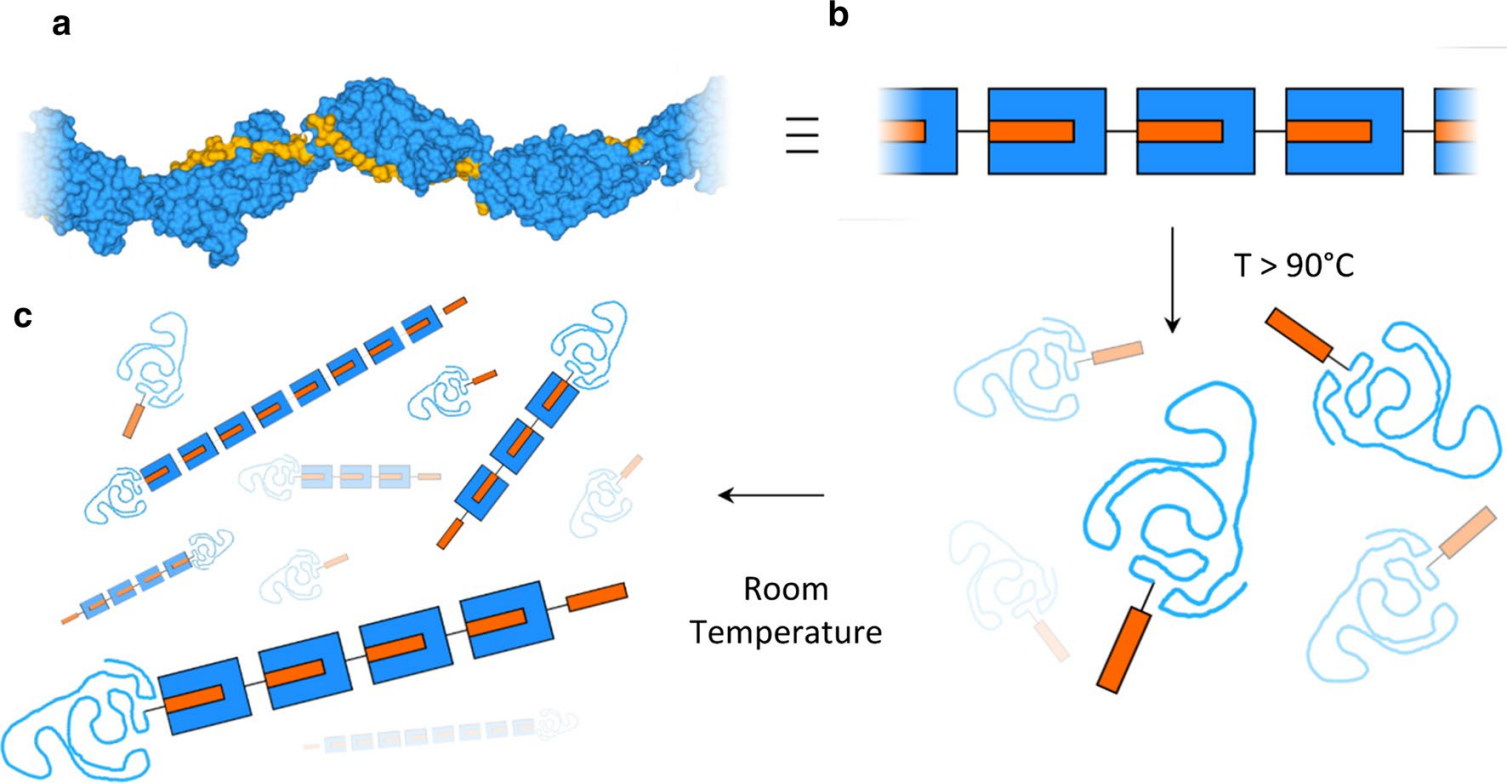
Structure and reversible thermal unfolding of Caf1 polymers. **a** Model of a segment of Caf1 polymer (generated from PDB entry 1P5U). The N-terminal donor strands (coloured orange) are inserted into the acceptor cleft of adjacent subunits. Protein structures were visualised using the CCP4MG molecular graphics package (McNicholas et al. 2011). **b** Cartoon depicting the reversible thermal unfolding of Caf1 polymers. When heated above the subunit melting temperature (*T*_m_ > 86 °C) the subunits (blue) thermally unfold with concomitant decomplexation of the N-terminal donor strands (orange), causing Caf1 to depolymerize into its unfolded monomeric form. When the subunits are incubated at room temperature they subsequently refold with donor strand recomplexation and concomitant repolymerisation, with a fraction of unfolded monomer still present (**c**)

Capsular antigen fragment 1 (Caf1) is an oligomeric protein consisting of 15 kDa monomer subunits that are non-covalently linked into a linear polymer chain (Fig. 1a). The protein belongs to the chaperone–usher class of bacterial pilins (Waksman and Hultgren 2009; Zav'yalov et al. 2010), which polymerise at the surface of *Yersinia pestis* bacteria to form long chains [up to 2 µm in length (Soliakov et al. 2010)] that protect the bacteria from the host immune system (Du et al. 2002). Each subunit possesses an incomplete immunoglobulin-like fold, which must be completed through donation of an N-terminal donor strand from a second subunit into the hydrophobic acceptor cleft, and by linking subunits together in this way, a polymer is formed (Zavialov et al. 2003). The association constant (*K*_A_) of the subunit interaction is estimated to be at least 10^14^ M^−1^, with dissociation times in the order of billions of years (Yu et al. 2012; Zavialov et al. 2005), and the remarkable strength of these kinetically inert inter-subunit linkages bestows the Caf1 polymer with excellent stability towards extremes of pH and proteases (Chalton et al. 2006; Ulusu et al. 2017).

We recently reported (Dura et al. 2020); however, that, despite their high stability, the inter-subunit linkages can be completely dissociated by utilizing thermal protein unfolding (Fig. 1b). At its melting temperature of 86 °C (Chalton et al. 2006; Zavialov et al. 2005), the subunits of Caf1 unfold with concomitant decomplexation of the N-terminal donor strands, causing complete depolymerisation of the Caf1 chain. We speculate that heat perturbs the tertiary structure of the acceptor to the extent that it is no longer complementary to the N-terminal donor strand, resulting in decomplexation. Surprisingly, this thermal unfolding-depolymerisation process is reversible, and upon cooling the subunits were shown to refold with concomitant recomplexation of the N-terminal donor strand, reforming the polymer chain (Fig. 1c). Throughout this process, a large initial fraction of uncomplemented (and hence disordered) monomer is present, decreasing over time as the subunits refold and reassemble into oligomers, but still present in small amounts even at longer time scales (Dura et al. 2020). Remarkably, Caf1’s capacity for reversible thermal unfolding is maintained even when extensively cross-linked to form a hydrogel network and endows their hydrogels with the capacity to melt and then reform, i.e., undergo a reversible gel–sol transition. This feature of Caf1 presents new material possibilities, and we were able to successfully demonstrate hydrogel reshaping, welding and blending. It was even possible to encapsulate live cells inside the hydrogel network, thus avoiding the use of reactive chemical cross linkers or expensive biorthogonal chemistries (Dura et al. 2020).

Macroscopic material properties depend critically on polymer molecular weight, and thus to further exploit the potential of the reversible unfolding feature of the Caf1 polymer in material development, it is important to better understand the nature and kinetics of the repolymerisation process. Indeed, our previous work (Dura et al. 2020) showed that the repolymerised form of Caf1 was of shorter lengths than the original bacteria-synthesized material, and an improved understanding of the repolymerisation process is likely to lead to the development of approaches to control of the lengths of the repolymerised forms of Caf1.

Insight to the kinetics of the Caf1 refolding process can be obtained from circular dichroism (CD) spectroscopy, which allows for the real-time observation of changes in protein tertiary structure during refolding. However, CD measurements do not provide direct information as to the changes in polymer molecular weight occurring during repolymerisation, the key information required to understanding any polymerisation process. In principle, the evolution of polymer molecular weight with time might be observable by size exclusion chromatography, but it is difficult to obtain quantitative molecular weight data for very large species. Transmission electron microscopy (TEM) can reveal the shape and length of the polymers but cannot make bulk, time resolved quantitative measurements. The highly complementary techniques of small-angle X-ray scattering (SAXS) and analytical ultracentrifugation (AUC) are widely used approaches for the study of biopolymers (Byron and Vestergaard 2015; Perkins et al. 2011) as they allow solute mass determination and shape characterisation either in terms of radius of gyration or frictional ratio/hydrodynamic radius, respectively. Furthermore, the almost instantaneous SAXS measurements available at modern synchrotron beamlines enable high resolution time-resolved studies (Franke and Svergun 2020). The SAXS method is sensitive to the mass and shape of the scattering molecule, allowing effective estimation of solute mass changes during the initial (fast) growth of Caf1 chains during repolymerisation. These measurements can be complemented with the determination of sedimentation velocity (SV) by AUC, which is a hydrodynamic method to estimate mass and shape of macromolecules. The changes in particle molecular weight and radius of gyration revealed uniquely by SAXS/AUC can thus be used to build a new understanding of rapidly polymerising biomolecules.

In this work we use SAXS to characterise the early, relatively rapid growth of Caf1 repolymerisation in solution. We then use AUC to investigate the behavior of the system in the latter stage of the repolymerisation, where the process is proceeding at a very slow rate. Methods to describe the structure and dynamic behavior of such protein polymers in solution are still evolving, and here we try to overcome these limitations by employing a number of different models to analyse the data. The results suggest that Caf1 repolymerisation proceeds in a linear fashion, with the absence of any lateral assembling, and with kinetics that are concentration-dependent. Importantly, over the first few hours (0–2 h) the polymer length was observed to grow relatively quickly, before continuing at a dramatically slowed rate. We discuss how this does not support our previously proposed in vitro polymerisation by a step-growth mechanism, and we speculate how these measurements hint at the possibility of a process more akin to chain growth, where one subunit at a time is added to the polymer chain. This analysis is the first of its kind for a bacterial fimbriae and our findings will help inform the use of SAXS and AUC in the in vitro repolymerisation of other protein-based polymeric species and, more broadly, the application of Caf1 polymers in the development of new biomaterials.

## Materials and methods

### Caf1 overexpression and purification

Caf1 was produced as described previously (Dura et al. 2020, 2018). Briefly, BL21(DE3) *E. coli* cells (NEB) were transformed with the pT7-COPΔR plasmid and grown in Terrific Broth (TB) media for 22 h at 35 °C. Cells were pelleted and Caf1 protein was extracted from the supernatant and flocculent layer using tangential flow filtration and gel filtration with a Captocore column (Cytiva). Samples were dialysed against nanopure water, lyophilised and resolubilised in phosphate buffered saline (Dulbecco PBS).

### Small-angle X-ray scattering (SAXS)

#### Sample preparation and repolymerisation procedure

Native Caf1^WT^ samples were used in our time-course SAXS measurements, and these were obtained as described above. The initial stock solution of the lyophilised protein was prepared at 13 mg/mL in PBS and the experimental sample concentrations (1.0, 2.0, 5.0, 10 mg/mL), subjected to repolymerisation were obtained by dilution of the initial stock in PBS.

The native Caf1^WT^ samples were heated to 90 °C for 5 min to achieve complete unfolding and monomerization then cooled on ice for one minute or left at room temperature for the remaining sample handling time. In general, a fast cooling method (i.e., on ice) was used to trigger the repolymerisation process, unless slow cooling (at room temperature) is specifically mentioned. The ice cooling procedure was expected to provide more efficient repolymerisation of Caf1 after thermal denaturation and was easier to standardise, while the room temperature cooling was considered to be less efficient for the repolymerisation process. Additionally, the fast cooling method was used in our previous study (Dura et al. 2020), so results obtained with this method are comparable to previously published data. Samples were centrifuged for 30 s at 20,238×*g* and placed into the Arinax sample handling robot (the bioSAXS robot, designed at EMBL) to continue repolymerisation process at 20 °C followed by timed SAXS measurements.

Some of the Caf1^WT^ samples used in our SAXS experiments went through the refolding process in-house over 24 h before the SAXS experiments. These samples were prepared using the fast cooling method described above. The concentration of these samples was 13 mg/mL during the repolymerisation process.

#### SAXS measurements

The small-angle X-ray scattering (SAXS) experiments were carried out at the beamline B21, Diamond Light Source (Didcot, UK) using a monochromatic X-ray beam (*λ* = 0.9466 Å) at the electron energy of 13.08 keV. The beamline was equipped with an EIGER 4M detector (Dectris, Switzerland) and the sample to detector distance was 2.696 m, covering a momentum transfer range of 0.0038 < *q* < 0.44 Å^−1^, where *q* = (4*π*sin*θ*)/*λ* and 2*θ* is the scattering angle. The beam was focused on the detector, the beam size was 1 mm (horizontal) × 0.5 mm (vertical) at the sample position and 0.008 mm (horizontal) × 0.07 mm (vertical) at the focal point. The protein sample (50 μL) was injected into a quartz flow-through capillary with a diameter of 1.6 mm and a thickness of 10 μm. The experimental temperature was kept at 20 °C.

The time-course SAXS measurements for each Caf1 concentration series were carried out by sequentially collected scattering curves of the same thermally denatured Caf1 specimen. The Caf1 material required to complete a single concentration time-course series was placed into the Eppendorf-holding rack of bioSAXS sample changer and delivered by the robotic system in standard aliquots (50 μL) to a quartz cell capillary inside the robot for X-ray scattering measurements. The measurements were carried out in the batch mode. During the initial rapid stages of Caf1 oligomerization (within the first hour after the native polymer monomerization) the time interval between consecutively collected scattering curves was governed by the time required to clean the sample-containing capillary and fill it with the next sample aliquot (1.5 min) inside the bioSAXS robot and safely exit the beamline hutch (7.5 min) before starting the data collection time course and obtaining the first data point. Thus, the first timepoint data were collected 7.5 min after the sample was heated and cooled and all consecutive measurements were made each 1.5 min during the first hour of data collection. These time intervals were determined experimentally before starting the actual time-course measurements. The same PBS solution was used in all measurements for all concentration series, the blank scans were recorded before and after first hour of uninterrupted sample injections in batch mode and, also, the buffer blanks were measured before and after the sample at later stages of the time course (3 h apart). Overall 30 blank scans were collected.

#### SAXS data analysis

Raw SAXS 2-D images were processed with the DAWN package (https://dawnsci.org/), the processing pipeline available at the beamline to produce normalised, integrated 1D un-subtracted SAXS curves. Caf1 behaviour under the beam was carefully monitored for radiation damage and the optimal number of frames for averaging was determined as 10 frames with 1 s exposure per measurement. The background subtraction, averaging of the data and determination of the scattering parameters in both reciprocal and real space along with molecular mass were executed in the program ScÅtter (http://www.bioisis.net). The form-factor modelling of the scattering data (*I*(*q*)) was carried out using the SasView package (http://www.sasview.org/). SAXS contrast for Caf1 and buffer solution was calculated using the SASSIE contrast calculator module (Sarachan et al. 2013). The polydispersity distributions were not included in the data analysis, thus all fitted parameters should be considered as mean values for the whole system. Thus, the molecular mass of the species estimated in our experiments was a weight average number <*M*> _*w*_ and the radii of gyration obtained from slope of the scattering curve were the higher momentum median values (also known as *z*-average) ${<{R}_{g}>}_{z}$ (Glatter et al. 1982).

The SoMo–SAS simulation module (Brookes et al. 2013) incorporated into the package SoMo (Brookes et al. 2010a, b; Rai et al. 2005) was utilised to calculate *P*(*r*) distributions for different oligomeric models of Caf1. The coordinates for the models were generated by the program Swiss PDB Viewer (Guex et al. 1999; Guex and Peitsch 1997) using the atomic coordinates for Caf1 dimer (Zavialov et al. 2003) (PDB accession code 1P5U).

##### Radii of gyration and mass determination

(i) The radius of gyration <$R$_*g*_>_*z*_ for every scattering curve (i.e., a single timepoint measurement) was estimated using the Guinier approximation (Guinier et al. 1955) which allows the evaluation of <${R }$_*g*_>_*z*_ at low *q* values (*q* × <$R$_*g*_> _*z*_ < 1.3) and could be determined from linear approximation in the following form: $\mathrm{Ln}I\left(q\right)\cong \mathrm{Ln}I\left(0\right)-\frac{{R}_{g}^{2}}{3}{q}^{2}$, where *I*(*q*) is the scattering intensity; *I*(0) is forward scattering intensity. Furthermore, the Guinier analysis could be enhanced using the Guinier peak analysis (GPA) procedure (Putnam 2016) which represents a modified form of Guinier approximation by multiplying both sides of the Guinier approximation by *q* as follows: $\mathrm{Ln}\left[qI\left(q\right)\right]=\mathrm{Ln}\left[I\left(0\right)\right]+\frac{\mathrm{Ln}\left({q}^{2}\right)}{2}- \frac{{R}_{g}^{2}}{3}{q}^{2}$. The dimensionless version of GPA (*qR*_*g*_*I*(*q*)/*I*(0) versus (*qR*_*g*_)^2^) is implemented in the program ScÅtter. The GPA characteristic peak position allows the validation of *R*_*g*_ values in complex systems when the Guinier region of scattering curve is represented by a small number of data points.

Alternatively, the value of <$R$_*g*_>_*z*_ could be evaluated from indirect Fourier transform (IFT) *P*(*r*) distance distribution function: ${<{R}_{g}>}_{z}^{2}=\frac{\underset{0}{\overset{{D}_{\mathrm{max}}}{\int }}{r}^{2}P(r)\mathrm{d}r}{2\underset{0}{\overset{{D}_{\mathrm{max}}}{\int }}P(r)\mathrm{d}r}$, where $P\left(r\right)=2{r}^{2}{\int }_{0}^{\infty }I(q)\frac{\mathrm{sin}(2\pi qr)}{2\pi qr}\mathrm{d}r$, with *r* denoting a distance within the scattering object, and *D*_max_ denoting the maximal distance of the scattering object (Glatter et al. 1982). In contrast with the Guinier approximation, the <*R*_*g*_> _*z*_ determined from IFT is calculated from the whole scattering curve.

Highly asymmetric objects, such as rods, can be characterised by the cross-sectional radius of gyration (*R*_c_) along with the general value of *R*_*g*_, describing the object as a whole. *R*_c_ characterises the shape of object in the cross section, and could be obtained from the intermediate Guinier region of the scattering curve (Glatter et al. 1982) in the following form: $I\left(q\right)= \frac{I(0)}{q}\mathrm{exp}\left[-\frac{{q}^{2}{R}_{\mathrm{c}}^{2}}{2}\right]$. This equation could be linearized as Ln[*qI*(*q*)] versus *q*^2^ and the slope of the straight line is proportional to *R*_c_. In our experiments the linear approximation was considered in the *q-*range of 0.0055–0.06 Å^−1^, satisfying the requirement: $\frac{2\pi }{L}<q{R}_{\mathrm{c}}<1$, where *L* is the length of Caf1 dimer (116 Å), the smallest folded oligomeric form of Caf1, roughly satisfying the requirement for an elongated particle *D*_max_/*d*_crossection_ > 2.5 (Glatter et al 1982) (the axial ratios for the dimer model are: *X*:*Z* = 3, *X*:*Y* = 2.4). In our experiments the cross-sectional radius of gyration was determined as a *z*-average value. Objects with a circular cross section, *R*_c_ can be converted to the geometrical radius (*R*) as $R=\sqrt{2}{R}_{\mathrm{c}}$.

The *Z*-average value of the radius of gyration could also be evaluated if the scattering curve is modelled with a known form factor. Thus, if a fractal model (Teixeira 1988) is used, the radius of gyration could be evaluated as ${<{R}_{g}>}_{z}^{2}=\frac{D(D+1){\xi }^{2}}{2}$, where *D* is fractal dimension and *ξ* is the correlation length (the average distance between entanglement points in a flexible polymeric chain) of the polymeric chain (Teixeira 1988).

In the case of the flexible cylinder model, the radius of gyration could be estimated as: ${<{R}_{g}>}_{z}^{2}=\frac{{a}^{2}N}{6}$, where *a* is the Kühn length (statistical length or twice the persistence length) and *N* is the number of subunits (Flory and Volkenstein 1969). In this present work all form factors were modelled using the SasView package (https://www.sasview.org/).

The weight-averaged mass of re-polymerising Caf1 for every timepoint was estimated using a volume-of-correlation (*V*_c_) invariant (Rambo and Tainer 2013) in the following form: $M={\left[\frac{1}{c}\frac{{V}_{\mathrm{c}}^{2}}{{R}_{g}}\right]}^{1/k}$, where the empirical constants are *c* = 0.1231 and, in the case of proteins; *k* = 1. Volume-of-correlation could be expressed as ${V}_{\mathrm{c}}=\frac{I(0)}{\int qI(q)\mathrm{d}q}$, therefore, $= \frac{1}{c}\times \frac{{I(0)}^{2}}{{R}_{g}}\times \frac{1}{{\left[\int qI(q)\mathrm{d}q\right]}^{2}}$. The experimentally determined values for the molecular weight were the weight average numbers.

Determination of *V*_c_ and, consecutively, the mass was performed using the program ScÅtter. The average number of subunits in oligomeric chain could be determined as <*N*> _*w*_ =  <*M*_*w*_^exp^>/*M*_0,_ where *M*_0_ is known mass of the monomer.

### Analytical ultracentrifugation sedimentation velocity (AUC-SV)

#### Sample preparation and refolding procedure

Solutions of post-denaturation Caf1 at concentrations of 1, 2, 5 and 7.5 mg/mL were prepared using lyophilised Caf1 in PBS. Monomeric Caf1 was obtained by heating the sample to 90 °C for 5 min. The repolymerisation process was triggered by cooling the samples at 4 °C. The repolymerised material was examined within the next 72 h after monomerisation followed by repeated measurement of the same sample stored at 4 °C. The repolymerised samples were diluted with relevant buffer prior to AUC experiments to concentrations between 0.2 and 0.4 mg/mL.

#### Data collection and analysis

Sedimentation velocity (SV) experiments were carried out in a Beckman Coulter Palo Alto, CA, USA ProteomeLab XL-I analytical ultracentrifuge using interference optics. All AUC runs were carried out at a rotation speed of 20,000 rpm at 20 °C using an 8-hole An-Ti50 rotor and double-sector aluminium-Epon centrepieces. The sample volume was 400 µL and the sample concentration was in range of approximately 0.2–0.4 mg/mL. Every repolymerised Caf1 sample was examined at two concentrations, at least, to ensure the reproducibility of the results. Caf1 partial specific volumes $\overline{v}$ were calculated from the protein amino acid sequence, using the program SEDNTERP (Laue et al. 1992). The density and viscosity of the experimental buffer (Dulbecco’s PBS) was also calculated using SEDNTERP. Sedimentation velocity profiles were treated using both the size-distribution *c*(*s*) and size-and-shape *c*(*s*,* f*/*f*_0_) models implemented in the program SEDFIT (Brown and Schuck 2006; Schuck 2000). The resulting distributions were also converted to standard conditions (*s*_20,*w*_). To determine the mass of each species, the *c*(*s*) distribution was converted either to a *c*(*M*) distribution or *c*(*M*,* f*/*f*_0_), respectively. Each peak on the distribution plot was integrated to obtain the weight-averaged values for sedimentation coefficient and molecular mass.

## Results

### The early stages of Caf1 repolymerisation can be observed by SAXS

**Fig. 2:**
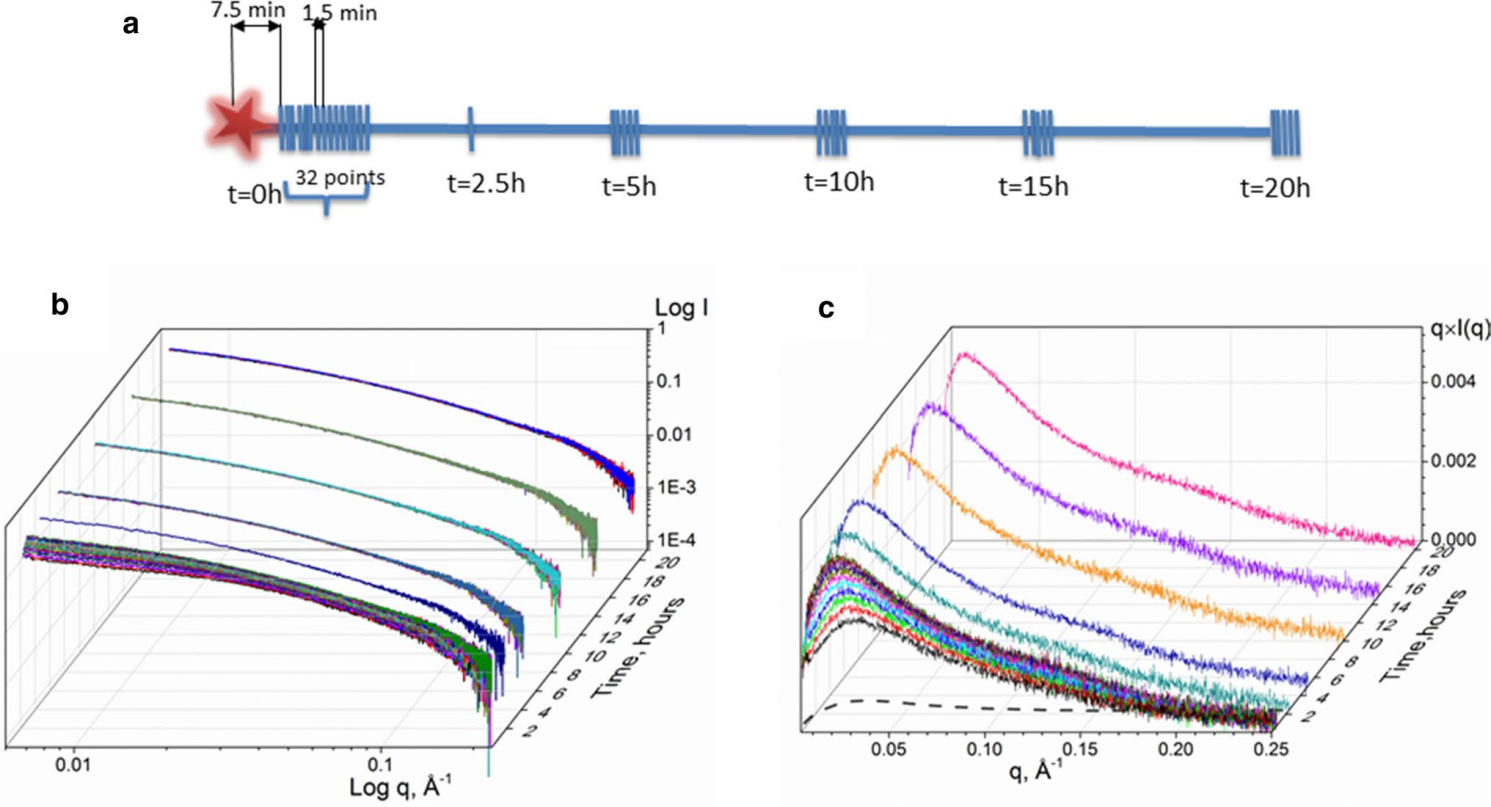
Experimental design of SAXS measurements for the Caf1 post-denaturation repolymerisation time course (**a**); a set of resulting scattering curves (the sample concentration was 5 mg/mL) in reciprocal space (**b**); and selected scattering curves from the same set represented as total scattering intensity versus scattering angle (**c**). These curves are shown together with the hypothetical total scattering intensity from a Gaussian coil (*R*_*g*_ = 41.3 Å, *c* = 5 mg/mL) (dashed line) which simulates the scattering of a disordered Caf1 monomer at zero time

Samples of native-Caf1 [the prefix native indicates this sample was obtained directly from the bacterial biosynthesis, consisting of chains averaging ~ 100 subunits (Soliakov et al. 2010)] at a selection of concentrations (1.0, 2.0, 5.0, and 10 mg/mL) were thermally unfolded and then cooled to allow refolding with concomitant repolymerisation. SAXS data were collected for each sample at the timepoints shown in Fig. 2a. An illustrative example of a set of scattering curves obtained at 5.0 mg/mL in reciprocal space is shown in Fig. 2b. The same dataset was converted to the total scattering intensity plot *q* × *I*(*q*) versus *q* (Fig. 2c) demonstrating the increase in total scattering intensity with time, particularly during the first hour of repolymerisation. The *q* × *I*(*q*) estimate for a hypothetical Gaussian coil simulating scattering of disordered Caf1 monomer (*R*_*g*_ = 41.3 Å, 5 mg/mL) at zero time was made to show the relatively low contribution that results from the disordered monomer (Fig. 2c). The generation of scattering curve for hypothetical Gaussian coil is described in details in Supplementary Materials and illustrated in Fig S1a, b.

### Intramolecular *P*(*r*) distance distribution

**Fig. 3:**
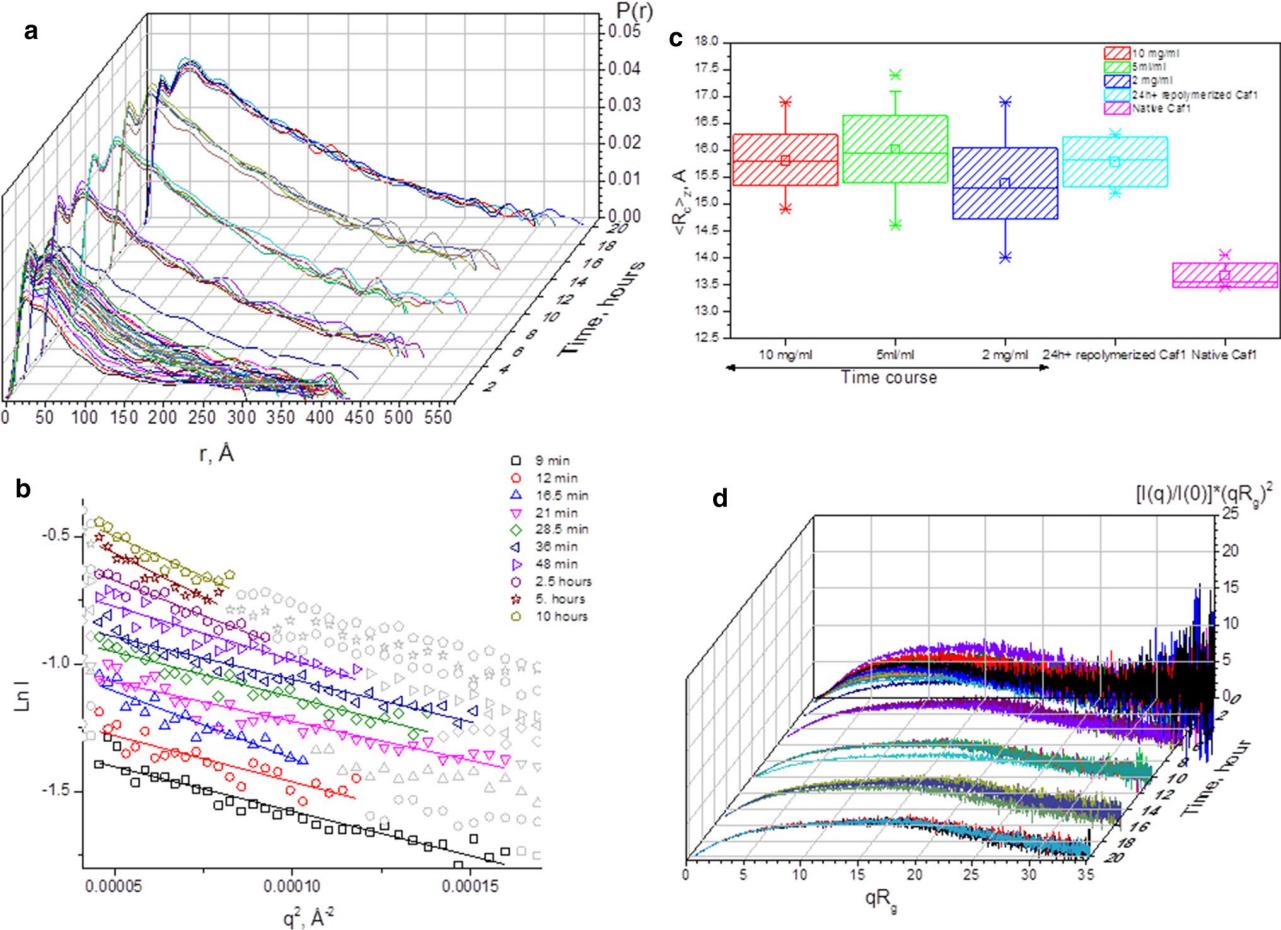
SAXS standard plots. **a** *P*(*r*) distributions show an increase in scattering particles’ maximal distance and similar positions for two main peaks on the distribution during the time course (the sample concentration was 5 mg/mL). **b** Guinier plot for the time-course sample at the concentration of 5 mg/mL. **c** Values of cross-sectional radius of gyration <*R*_c_>_*z*_ do not substantially change with concentration and time for the repolymerazing sample. A smaller value of *R*_c_ was obtained for native Caf1^WT^. **d** Shape of a dimensionless Kratky plot suggests multimeric scattering particles elongating with time (shown for the sample concentration of 5 mg/mL)

Scattering curves obtained in reciprocal space *I*(*q*) could be converted to real space using indirect Fourier transformation (IFT) and represented in the form of a distance distribution function *P*(*r*)) (Fig. 3a). *P*(*r*) distributions were calculated using ScÅtter (https://www.bioisis.net). The Legendre method was used for indirect Fourier transform as this was most suitable for elongated molecules. For transformation, *q*_min_ was set to 0.0067, 0.0085 and 0.011 for 5 mg/mL, 2 mg/mL and 1 mg/mL, respectively, and kept fixed through the time course. The value of *q*_max_ for each dataset was set at the point when total scattering intensity was zero (*q* × *I*(*q*) = 0), and was also evaluated in ScÅtter.

The shape of the *P*(*r*) functions resulting from the repolymerisation time course suggests the formation of continuously elongating species with two clearly visible common peaks observed at shorter distances throughout the whole time course. The first peak (with a maximum at ~ 20 Å) represents distances within the ~ 60 × 30 Å monomeric unit. The following peak (at ~ 44 Å) is relevant to the correlation distances to the second neighbouring subunit. As the repolymerisation of Caf1 proceeds, this peak shifts slightly towards longer distances, as would be expected if inter subunit correlations increase. Correlations to more distant subunits are poorly resolved on account of the inter-subunit flexibility which results in the smoothing of the *P*(*r*) function. These findings agree with published results for a model flexible multimeric system, albeit significantly smaller than Caf1 (Bernado 2010). The observed irregular ripples of *P*(*r*) at larger distances are probably the result of oversampling of the scattering data in the indirect Fourier transform as a consequence of the high dispersion of the scattering at high angles. Theoretical *P*(*r*) distributions calculated for the examples of multimeric linear rigid Caf1 structures (shown in Fig. S2) show overall similarity with experimentally obtained *P*(*r*) distributions*.* Thus, these observations indicate that the Caf1 subunits likely repolymerise to form growing flexible oligomeric species.

### Guinier analysis

The Guinier plot is one of the important standard plots in SAXS data analysis which allows the determination of the radius of gyration (*R*_*g*_) of the scattering object (Glatter et al. 1982). The characteristic linear approximation of the scattering curve could be made in the low-*q* region, allowing determination of the general value of *R*_*g*_*.* TEM images of samples of repolymerized Caf1 (Dura et al. 2020), suggest that the species formed could be polydisperse and contain variable numbers of Caf1 monomers, therefore, only a z-average value of radius of gyration (${<{R}_{g}>}_{z}$), characterising the whole system could be determined (Glatter et al. 1982). The linear approximation Ln*I*(*q*) versus *q* used the same starting point (*q*_min_) as the IFT above (*q*_min_ was set to 0.0067, 0.0085 and 0.011 for 5 mg/mL, 2 mg/mL and 1 mg/mL, respectively—the linear approximation for the 5 mg/mL time-course set are shown in Fig. 3b). All Guinier approximations were run through the Guinier peak analysis procedure to ensure that the region selected for linear approximation fits the shape of the Guinier peak (Putnam 2016). Several illustrative examples of the Guinier peak analysis are shown in Fig. S3.

The TEM images obtained in our earlier work (Dura et al, 2020) indicated the formation of elongated structures, thus the determination of <*R*_c_>_*z*_ has to be included in the characterization of repolymerising Caf1. The Guinier plot in the so-called “intermediate-*q* region” (Ln(*qI*(*q*)) versus *q*^2^) (Glatter et al. 1982) at each timepoint in the repolymerisation process (Fig. S4a), along with the native Caf1^WT^ (Fig S4b) allows the estimation of the cross-sectional radius of gyration (<*R*_c_>_*z*_), a highly informative parameter for elongated multimeric molecules (Sund et al. 1969). The <*R*_c_>_*z*_ value for repolymerised Caf1 was found to be 15.8 ± 0.2 Å and was observed to remain constant during the time course for all concentrations examined for repolymerised samples (Figs. 3c and S4c). This observation suggests that the growing polymer remains linear in nature and suggests no lateral association. Analysis of a sample of native-Caf1^WT^, on the other hand, revealed a slightly smaller cross section (13.7 ± 0.06 Å), which translates into a geometrical radius of 19.2 Å (Fig. 3c). The hydrodynamic model built on the basis of Caf1 monomer atomic coordinates (PDB accession code 1P5U (Zavialov et al. 2003), reveals a prolate ellipsoid with the dimensions of 57.3 × 38.3 × 37.3 Å (hydration included); therefore, the geometrical radius (*R*) and *R*_c_ for the model can be estimated to be 18–19 Å and 13 Å, respectively*.* This is in good agreement with our experimentally determined value of <*R*_c_>_*z*_ (13.3 Å) for native Caf1^WT^ (the relationship between *R* and *R*_c_ for the circular cross section can be found in the Materials and Methods section). Thus, repolymerized Caf1 samples demonstrate an excess of 3 Å in <*R*_c_>_*z*_ values compared to native Caf1^WT^ chains (Fig. 3c). This excess was also observed in the samples repolymerising for the period over 24 h. We believe that the presence of disordered monomer and/or propensity to further oligomeric growth may cause this difference.

### Dimensionless Kratky plot

The scattering curves obtained at each timepoint during Caf1 repolymerisation were processed to obtain the relevant dimensionless Kratky plots ((*qR*_*g*_)^2^ × *I*(*q*)/*I*(0) versus *qR*_*g*_) to assess the cumulative conformation of the polymeric chains (Durand et al. 2010). The dimensionless Kratky plot for a time-course set of curves (shown in Figs. 3d and S5 for the 5 mg/mL sample concentration series) is neither exactly parabolic nor hyperbolic, the shapes characteristic of folded/globular particles or random walk polypeptide chains, respectively (Receveur-Brechot and Durand 2012). Instead, it reveals an asymmetric broad peak extending towards high *qR*_*g*_ values with time*.* This suggests that growing flexible Caf1 polymers elongates with time and in agreement with the results of Bernado’s theoretical work (Bernado 2010). The dimensionless Kratky plot for the data collected at the earlier stages of the time course, up until the 10th–15th timepoint (depending on the sample concentration), shows a slight elevation above zero on *qR*_*g*_-axis (shown in Fig. S5, top panel). This could be caused by some amount of free disordered monomer that remains at the earlier stages of repolymerisation time course and as the repolymerisation progresses, the folded polymeric chains start to dominate on the plot in form of fully converged curves (Fig. S5, bottom panel).

### Evolution of Caf1 chain sizes during repolymerisation

**Fig. 4:**
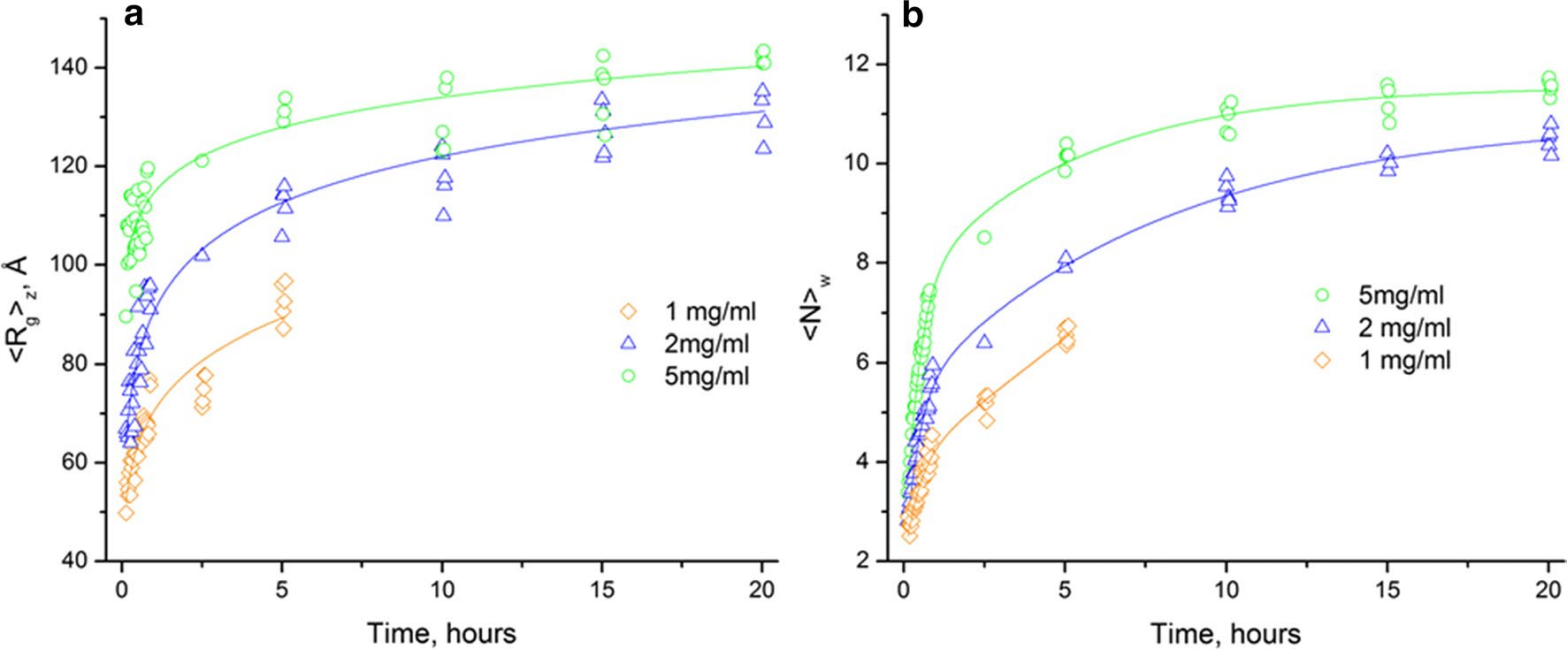
Increase in z-average values of radius of gyration (**a**) and in weight-averaged number of chain subunits (**b**) of repolymerised Caf1 during the time course. The concentration of repolymerized Caf1 affects the rate of increase. <*R*_*g*_>_*z*_ values were obtained from *P*(*r*) distribution and the average number of subunits (<*N*>_*w*_) in the repolymerised chain was calculated on the basis of <$M$>_*w*_ values evaluated using the Guinier approximation. The samples at 1 mg/mL were examined for the fast stage only

As described, SAXS can provide unique information about the Caf1 repolymerisation process by allowing us to observe how the radius of gyration of the whole system changes during repolymerisation. Both approaches to determine radius of gyration (from Guinier approximation and IFT) gave very similar values for ${<R>}_{z}$ (Fig. S6). We observed a concentration dependency, with the growth rate in <*R*_*g*_>_*z*_ increasing with Caf1 concentration (Fig. 4a). The rate of growth in <$R$_*g*_>_z_ values decreases as the repolymerisation progresses. At the end of the data collection (20 h) the rate of increase was observed to be very slow.

The solute weight-averaged mass could be determined using a volume-of-correlation invariant obtained from scattering intensities (Rambo and Tainer 2013) and, finally, the weight-averaged number of subunits in polymeric chain (<*N*>_*w*_) as <*N*>_*w*_ =  <*M*^exp^>_*w*_/*M*_0_, where *M*_0_ is the mass of the monomer.

The increase in <*N*>_*w*_ versus time consists of a steep exponential section during the early phase (0–2.5 h) of the repolymerisation process and at the later phase (> 2.5 h) growth continues but at a significantly lower rate similarly to the changes in <$R$_*g*_>_*z*_ (shown in Fig. 4b for a repolymerising sample at 5 mg/mL).

### Form factor modelling

In our earlier work, the native Caf1 oligomer was captured on the TEM images as long chains which appear as a random walk polymers (Soliakov et al. 2010) forming wiggles and loops. Such behavior is suggestive of the extremely flexible nature of these bio-filaments. The form-factor modelling approach to analyze the small-angle scattering data for hydrogel-forming systems was demonstrated earlier (Draper et al. 2017; Fuentes-Caparrós et al. 2019). These developments could be relevant for Caf1 polymers to some extent, owing to Caf1’s ability to form a hydrogel (Dura et al. 2018). Thus, for the next step in this study we tried to fit Caf1 scattering curves to a number of form-factor models in the attempt to describe the scattering behavior of Caf1 chains analytically.

**Fig. 5:**
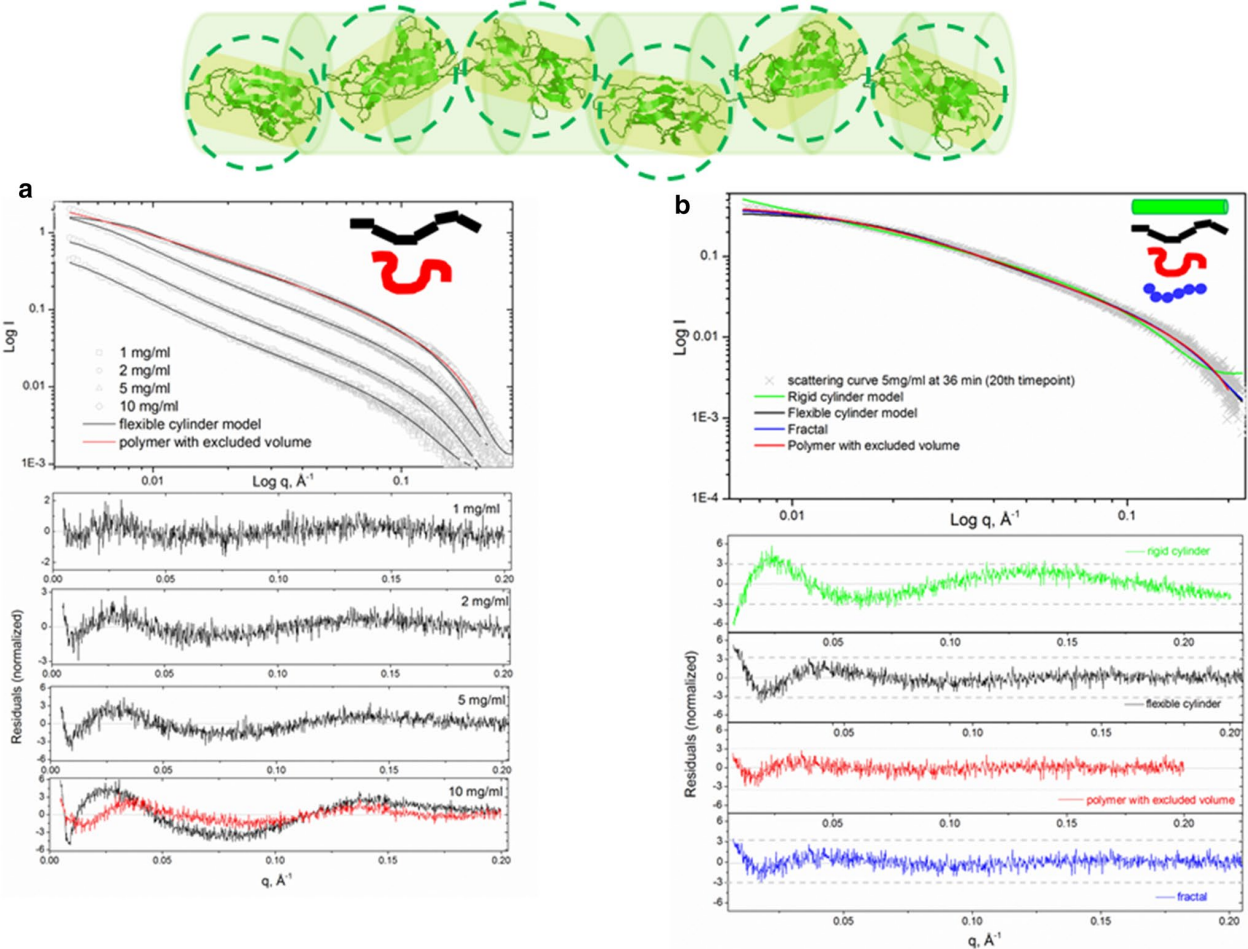
Linear model of Caf1 oligomer (cartoon in green-top panel) represented as various form-factor models: rigid cylinder (whole oligomer), flexible cylinder (every monomer or group of monomers in the chain is considered as fragments of flexible cylinder), and shape-independent mass fractal (spherical subunit monomers) and polymer with excluded volume—a basic model for scattering of various polymeric chains. The bottom panel shows an illustrative example of the experimental scattering curve fit to these models. The native Caf1^WT^ modelled as a flexible cylinder (black line) at 1 mg/mL, 2 mg/mL, 5 mg/mL and 10 mg/mL and a polymer with excluded volume (red line) at 10 mg mg/mL (**a**). Repolymerising Caf1 at 36 min post-denaturation, (sample concentration 5 mg/mL) modelled as a rigid cylinder (green line), flexible cylinder (black line) fractal (blue line) and a polymer with excluded volume (red line) (**b**)

In general terms, small-angle scattering of a particle (i.e., the scattering intensity function *I*(*q*)) in solution can be defined by the particle’s concentration (volume fraction), its contrast in solution and its form-factor scattering intensity function. In many cases, scattering curves produced by biomolecules and their ensembles in solution can be successfully fitted to known form factors calculated for geometrical bodies (Pedersen 1997). In the first instance we analysed the scattering curves of the native Caf1^WT^ using a flexible cylinder model (Pedersen and Schurtenberger 1996) which satisfactorily fitted our data [Fig. 5 bottom panel, (a)]. The length of about 3000 Å and radius of approximately 15 Å were in reasonable agreement with published earlier TEM findings for native Caf1^WT^ polymeric chains (Soliakov et al. 2010). The fitted value for the flexible cylinder radius is also in agreement with a hydrated Caf1 monomer model based on its high resolution structure (Zavialov et al. 2005). The value for the statistical length (Kühn length) of a native Caf1^WT^ oligomeric chain was found to be 170 Å, which is approximately three monomer lengths. This model fit the experimental data almost comprehensively except at very high sample concentrations (10 mg/mL) The model for a polymer with excluded volume (Hammouda 1993) fits the experimental data at this sample concentration slightly better due to accounting for the concentration effects in solution.

The repolymerisation process of Caf1 is a dynamic process since the shape of growing oligomers (and, consequently, the form-factor models describing them) may vary depending on the sample concentration, the length of the growing oligomer and the rate of growth. Therefore, several form-factor models were examined to analyze the time-course data. An illustrative example of the form factor model fits is shown in Figs. 5b and S7 representing the datafit and/or residuals by the following models (i) a flexible cylinder model, which was used to analyse the native Caf1^WT^ samples, (ii) a rigid cylinder model, which could be applicable for the earlier stages of growth when the chains are still short (about the statistical length of the polymer), (iii) a fractal model and (iv) a polymer with excluded volume model. The two latter models are shape-independent and are applicable to a flexible polymer (Capp et al. 2014). On the same note Caf1 growing oligomers in vitro may be modelled as a fractal system, where each Caf1 monomer joining the chain can be considered as a repeat building block. As the chain grows from monomer into a larger oligomer, its general geometrical features change, which can be described quantitatively in terms of fractal dimension (*D*). Folded globular proteins typically exhibit a value of *D* ~ 2.8 (Isogai and Itoh 1984), i.e., a 3D shape, while the behaviour of a long oligomer chain can be represented as a line (i.e., 1D shape).

**Fig.6:**
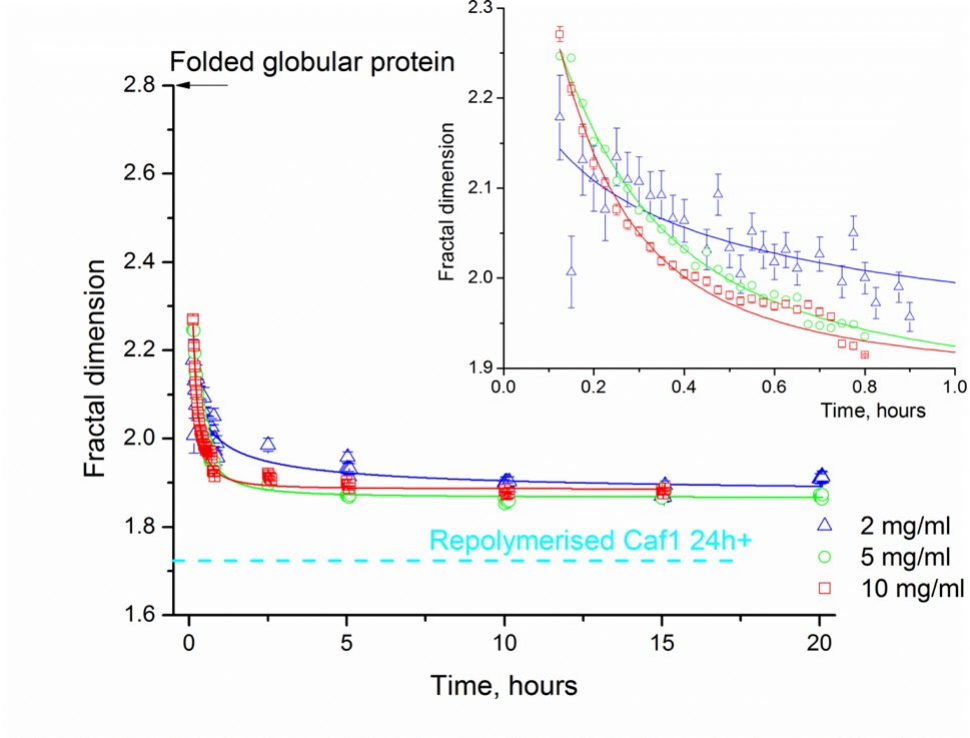
Changes in the geometry of a repolymerized Caf1 chain in terms of fractal dimension. Fractal dimension drops very quickly during the first hour of repolymerisation suggesting the formation of polymers in the form of wiggly lines *D* ≅ 1.9, ultimately approaching the *D* value of 1.72 detected for the Caf1 sample repolymerised for more than 24 h in advance (the concentration of repolymerising sample was 13 mg/mL, whereas this sample concentration in SAXS experiment was 6.75 mg/mL). The changes in fractal dimension during first hour of repolymerisation are shown in the insert. The data obtained for the low concentration (2 mg/mL) repolymerized material, which polymerizes slower than at the higher concentrations, demonstrates slower linearization, probably on account of a relatively large amount of disordered monomeric material being present during the polymerisation

A flexible polymeric chain is expected to have fractal dimensions varying between 2 and 1 as it entangles and wriggles (Dewey 1998). We anticipated that as the Caf1 chain grows in length during repolymerisation, *D* would decrease towards unity, the value anticipated for a flexible indefinitely thin cylinder (*L* >> *d*). A plot of *D* versus time for repolymerisation at a selection of concentrations (2.0, 5.0, 10 mg/mL) is presented in Fig. 6. At the first timepoint after the initiation of repolymerisation, the observed fractal dimension of the system was observed to be in the range of 2.25 suggesting the presence of low order oligomers, such as trimers or tetramers, for example. As the repolymerisation proceeds, *D* was observed to decrease rapidly then essentially plateau, tending towards a value of *D* ~ 1.72, detected for a Caf1 sample repolymerised more than 24 h in advance. The latter seemingly low value for *D* could be explained by the fact that this sample was repolymerising at much higher concentration (13 mg/mL) which results in the formation of a longer polymeric chain compared to the repolymerised Caf1 samples studied in the time course. Overall, these observations indicate that Caf1 repolymerizes to reform a linear species with no lateral self-association, i.e., its repolymerized form is identical to its original bacteria-synthesized form (Soliakov et al. 2010).

The fitted values of fractal dimension (between 2.3 and 1.7) (Fig. 6) for in vitro Caf1 polymerisation suggest that the polymer with excluded volume model could be also applied (Hammouda 1993). From the perspective of the <*R*_*g*_>_*z*_ values characterising the entire system, this model is quite similar in essence to the fractal model. The <*R*_*g*_>_*z*_ value derived from the polymer with excluded volume model is a parameter that is directly fitted to the scattering curve, while in the fractal model, it has to be calculated. Overall, all models give similar values for <*R*_*g*_>_*z*_, which are comparable to those obtained in the Guinier approximation/IFT (Fig. S7), with the exception of the <*R*_*g*_>_*z*_ values derived from the parameters of the rigid cylinder—those are in the agreement with others at early stages of repolymerisation only.

The residual plots (Fig. 5b, bottom panel, and Fig. S7) show that the flexible cylinder and both shape independent models possess a similar distribution of residuals, although the polymer with excluded volume model gives the smallest deviations. The rigid cylinder model gives a reasonable fit only at very early stages of repolymerisation (as shown in Fig. S7, top panel). A noticeable systematic deviation at very low scattering angles (*q* < 0.01) was present for all form-factor model fits, particularly for the scattering curves collected in first 10–20 min of the time course. Hence, it may be concluded that neither model gives an ideal fit to the data in this *q*-range. This outcome could be explained by a contribution of a small number of large species present in the rapidly oligomerising Caf1 solution at the beginning of the repolymerisation process, when the majority of the material is still rather small. As the repolymerisation progresses, this effect seemingly diminishes as the majority of the material acquires longer polymeric chains, thereby decreasing the degree of polydispersity in solution. It seems that the level of polydispersity in solution is dependent on the method used to initiate Caf1 repolymerisation, i.e., it is characteristic for thermally denatured Caf1 samples cooled on ice and then subjected to repolymerisation. In a contrast, the scattering curves produced by the slow repolymerising method (i.e., where the repolymerisation process was triggered at room temperature) are much better fitted by the form-factor models over the low *q*-range (Fig. S9). The latter samples demonstrate significantly slower time-dependent increase in mass and radius of gyration (Fig. S10).

### The latter phase of Caf1 repolymerisation can be observed by AUC

**Fig. 7:**
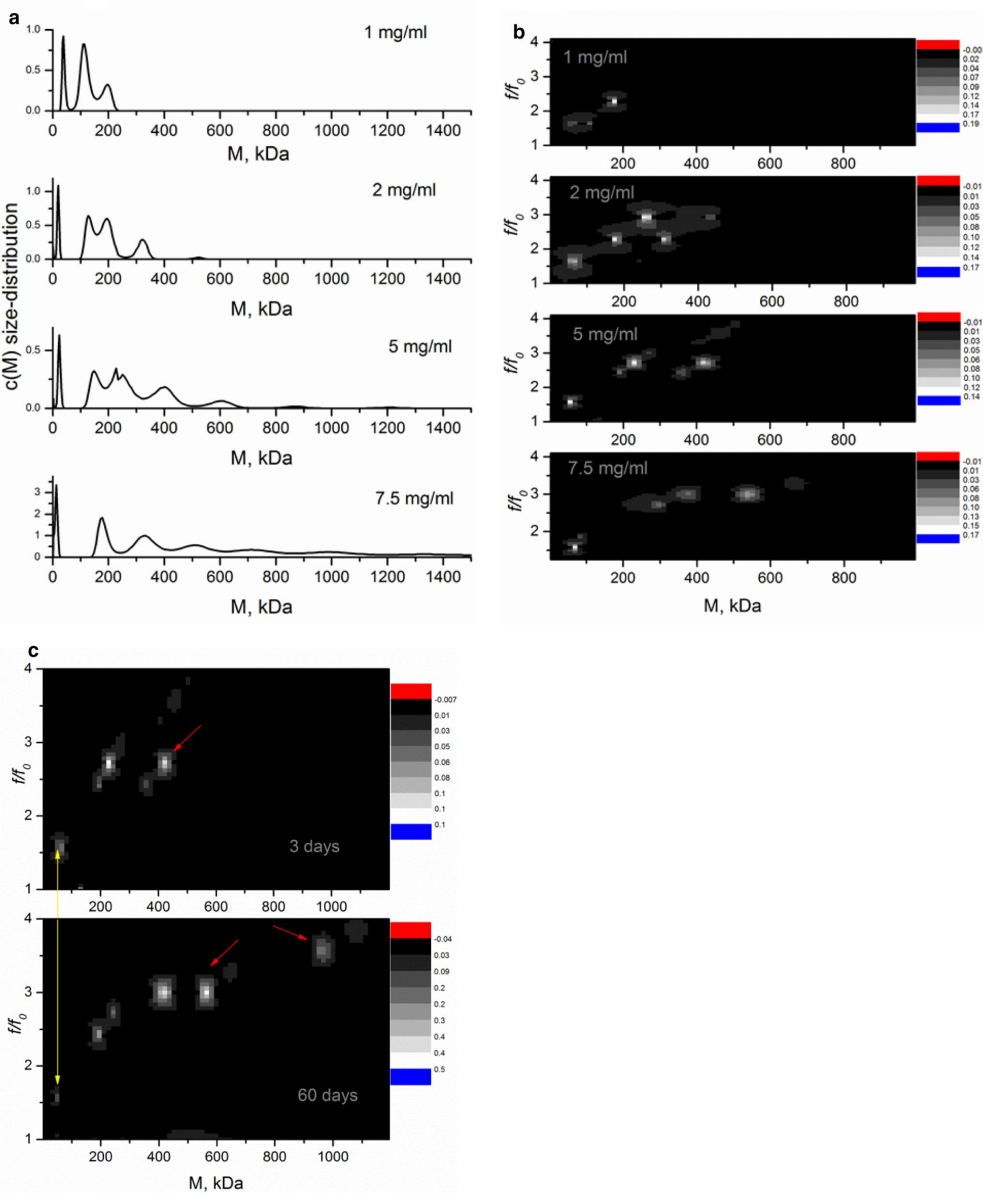
Repolymerised Caf1 samples analysed by sedimentation velocity analytical ultracentrifugation experiments (the rotation speed was 20,000 rpm) modelled as 1D *c*(*M*) (**a**) and 2D *c*(*M*, *f*/*f*_0_) (**b**) size distributions. Long storage time result in the appearance of heavier and longer species (marked with red arrows), while smaller species still exists (marked with yellow arrows) as seen from the 2D distribution *c*(*M*, *f*/*f*_0_) (**c**)

The SAXS studies indicated that after an initial fast growth phase, the rate of growth of Caf1 chains becomes significantly slower. Analytical ultracentrifugation sedimentation velocity can provide information about the chain lengths of repolymerized Caf1 in this latter slow growth phase, i.e., at significantly longer experimental time scales in comparison with SAXS experiments. Samples of native-Caf1^WT^ at 2.0, 5.0 and 7.5 mg/mL were thermally unfolded, cooled to 4 °C and left to repolymerize for 72 h (3 days) and then analysed by AUC. The solute distribution in an ultracentrifuge cell can be represented in the form of 1D *c*(*s*) or 2D *c*(*s*,*) (Brown and Schuck 2006; Schuck 2000). The latter is applicable for particularly complex heterogeneous systems, where * could represent properties of the biomolecule, such as *f*/*f*_0_,* M*, *D*, *R*_S_ (Brown and Schuck 2006; Chaton and Herr 2015)_._ Both 1D and 2D size-distribution models were applied to analyze sedimentation velocity profiles (the experimental data and fit are shown in Fig. S11a–d). The distribution of solute masses is shown in Fig. 7a (the corresponding distributions of sedimentation coefficients is shown in Supplementary material, Fig. S12a). The concentration of repolymerising sample seems to be an important factor, since the mass distributions broadened and shifted towards higher values as the concentration of repolymerised sample increased from 1.0 mg/mL to 7.5 mg/mL. The observed increase in mass was accompanied by frictional ratio changes from 1.8 to 2.9 in the 1D distribution suggesting an overall elongation of the molecules as the refolding concentration increases. To minimize the contribution of highly asymmetric shapes that result in the non-ideal behavior of the solute, it was important to dilute the solutions immediately prior to the experiment. Thus, the actual sample concentration in sedimentation velocity experiments was typically about 0.2–0.4 mg/mL; however, due to the extremely slow rates of chain growth, the sample dilution effect was unlikely to affect the observed masses. More detailed insight on the nature of repolymerized Caf1 was gained by moving from 1D distributions, where the values of *f*/*f*_0_ are average numbers for all observed species, to 2D distributions, where the parameters mass and shape were fitted simultaneously (Figs. 7b, S12b, d). This approach demonstrates clearly that the higher masses that are detected are characterized by higher *f*/*f*_0_ values, i.e., the chain becomes longer as the sample concentration increases. Thus, the largest detected species ranged from ~ 148.2 kDa at the Caf1 repolymerisation concentration of 1 mg/mL, to ~ 576.3 kDa at repolymerisation concentration of 7.5 mg/mL. Interestingly, the analysis of samples stored for 60 days show a more populated profile with a strong peak (Fig. 7c) at mass ~ 600 kDa (16% of total material) and less resolved peaks at 1MDa (8% of total material), indicating further repolymerisation beyond 3 days, when the maximal detected mass was ~ 400 kDa (20%) (marked in Fig. 7c with red arrows). The species with the mass of ~ 200 kDa were detected in both cases (marked with a yellow line) at 23% and 26% of the total, respectively. These observations indicate that Caf1 repolymerisation slowly continues beyond the initial 20 h window of the SAXS experiments, and suggests the terminal subunits of Caf1 oligomers remain ‘active’ and are capable of forming linkages with other monomers or chains, even at long reaction times. In all experiments, a fraction of small Caf1 oligomers (dimeric or trimeric size) were also detected alongside the larger polymers.

## Discussion

Repolymerized Caf1oligomers are considerably shorter in length than the native form of Caf1, but exhibit some variety in mass and shape. Therefore, analysis methods based on statistical conformation of the whole ensemble are particularly useful as shown in (Capp et al. 2014). However, most SAXS studies of oligomeric flexible systems were based on preliminary knowledge of the number of subunits within the oligomer as in (Bernado 2010, Capp et al. 2014) that help to enable modelling of the scattering intensity resulting from stochastic movements of the oligomeric chain, as implemented in EOM (Bernadó et al. 2007) or SASSIE (Perkins et al. 2011). An attempt to describe the indefinite repolymerization of Caf1 subunits in terms of mass fractals is probably the most justified approach, since this model is totally shape-independent. To the best of our knowledge, this model has not yet been applied to regular oligomeric growth. The fractal model only describes the conformational dynamics of the chain and does not provide any information about actual sizes of particles, however, combining this analysis with another approaches, such as Guinier/IFT, allows for a more complete characterisation of the system.

A key advantage of SAXS experiments in the study of the repolymerization of Caf1 is its capacity to passively observe the almost instantaneous determination of solute mass and shape; however, the measurements are limited by *q*-window of the camera length distances in bioSAXS instrument. We were thus able to monitor effectively the repolymerization for approximately the first 10 h before some oligomers likely became too long to be detected by the *q*-window of the measurement. Conveniently, it was possible to detect further growth on a much slower experimental time scale in sedimentation velocity experiment and trace the small oligomeric forms of Caf1 which could be “hiding” behind the dominant signal coming from larger oligomers in the SAXS experiments.

In contrast to SAXS, where detected masses are the weight averaged, analytical ultracentrifugation can, in principle, detect the mass for each individual oligomeric form. The method cannot describe the mass distribution of oligomeric species forming during first few hours of the repolymerisation, but it is effective approach for slow maturing oligomeric forms.

SAXS and AUC together provide experimental data that indicate how the Caf1 polymer grows with time during the repolymerization process. The experimental evidence obtained suggests that Caf1 repolymerization occurs in a linear fashion, with no evidence of lateral association or aggregation. This result is in agreement with published earlier TEM images of native and repolymerised Caf1 (Soliakov et al. 2010; Dura et al. 2020) which reported no observation of Caf1 chain branching. The rate of repolymerisation, as determined by SAXS, appears to show a concentration dependency (in the range 1.0–5 mg/mL), which is in agreement with earlier CD studies (Dura et al. 2020). Irrespective of the model used to fit the SAXS data, the oligomeric growth shown in Fig. 4b displays two key features: a relatively fast exponential growth over the initial stage (0–2.5 h) and then a much slower linear growth over the remaining duration of the experiment. The donor–acceptor nature of the Caf1 monomer unit suggests that its in vitro polymerization likely proceeds by a step growth mechanism, where the monomers react together in a stepwise fashion to form shorter oligomers that continue to react to form longer oligomers. However, with classical step growth polymerizations a linear growth of molecular weight with time can be expected (Stille 1981). Thus, the observations made by SAXS and AUC are not consistent with the idea of Caf1 polymerizing by step-growth process. We thus considered the possibility that the initial exponential growth in molecular weight may arise on account of the operation of an isodesmic polymerization mechanism (Zhao and Moore 2003). An isodesmic polymerization can be considered the supramolecular analogue of a classical step growth polymerization in which monomers reversibly interact in a stepwise fashion to form short oligomers that then continue to react to form longer oligomers. However, an isodesmic polymerization would be expected to reach equilibrium (i.e., molecular weight stops growing and becomes constant), but that does not appear to be the case as the AUC experiments indicate observed small increases in molecular weight occurring, even after several days, suggesting an isodesmic mechanism also is not operational. The kinetic plot in Fig. 4b is also not consistent with nucleation growth (Zhao and Moore 2003), a well-known protein polymerization mechanism in which there is a relatively slow nucleation phase followed by rapid polymerization, eliminating the possibility of this mechanistic pathway.

In vivo, native-Caf1^WT^ polymer biogenesis follows the conserved chaperone–usher pathway used by Gram-negative bacteria to construct a variety of surface polymeric structures (Waksman and Hultgren 2009; Zav'yalov et al. 2010). This process involves the sequential addition of subunits onto the growing end of the polymer chain, and can be considered to resemble a chain-growth polymerization mechanism (McGrath 1981), where monomer units add one at a time onto the end of a growing chain. The evolution of molecular weight with time observed in our in vitro experiments does appear to present similarities with that observed for chain growth polymerizations. Early in a chain growth process when monomer is present in a large excess in relation to chain ends, molecular weight increases quickly. When the concentration of monomer decreases to become similar to that of active chain ends, the rate of molecular weight growth slows down, and then becomes increasingly slower as the concentration of monomer continues to decrease. It is tempting to think that such a process might be operating in the in vitro polymerization process. This, however, would require a plausible mechanism as to how Caf1 subunits can add one at a time onto the end of a chain in the absence of chaperone and usher proteins that are required to mediate the in vivo polymerization observed in Gram-negative bacteria, which is beyond the scope of this current work. Experiments ongoing in our laboratories hope to shed further light on the mechanism of in vitro Caf1 polymerization.

## Conclusion

In this work we have demonstrated how SAXS and AUC can be utilized to study the in vitro repolymerization of Caf1. SAXS data could be used to determine the evolution of polymer growth with time, and reaffirms the concentration dependency of the rate of repolymerization. SAXS data analysis of a 20-h time course was based on a combination of both shape-dependent and shape-independent approaches giving an insight on fast initial phase of Caf1 repolymerization. The AUC method was employed to observe evidence of the process at longer time scales, where there continued to be a very slow growth of the oligomers. A key finding of this work was that the observed evolution of polymer molecular weight with time, where weight grows rapidly (0–2.5 h) and then far more slowly, was not consistent with that of classical step growth or isodesmic polymerizations. Intriguingly, the evolution of molecular weight might be better explained by a chain growth mechanism, whereby subunits attach one at a time onto the end of a chain. Such a polymerization process may bear similarities to the conserved chaperone–usher pathway used in vivo, where the polymerization process is mediated by chaperone and usher proteins to sequentially attach subunits one at a time one the C-terminus of the growing polymer.

This work shines new light on the Caf1 repolymerization mechanism, which was previously thought to involve irreversible association of subunits and proceed through a conventional step-growth mechanism. This improved understanding of the Caf1 repolymerization process will allow the development of methods to better control polymer growth, and ultimately, better control the properties of Caf1-based materials.

## Supplementary Information

Below is the link to the electronic supplementary material.

Supplementary file1 (DOCX 5468 KB)
