# Supplementary material for: Probing the oligomeric re-assembling of bacterial fimbriae in vitro: a small-angle X-ray scattering and analytical ultracentrifugation study
Source: Eur Biophys J. 2021 May 4;50(3-4):597–611. doi: 10.1007/s00249-021-01543-3 (PMC8190007; doi:10.1007/s00249-021-01543-3)
Supplement: Supplementary file 1 — Supplementary file1 (DOCX 5468 KB) [file 249_2021_1543_MOESM1_ESM.docx]

European Biophysics Journal

Supplementary Materials

**Probing the oligomeric re-assembling of bacterial fimbriae *in vitro*: a small-angle X-Ray scattering and analytical ultracentrifugation study**

Alexandra S. Solovyova, Daniel T. Peters, Gema Dura, Helen Waller, Jeremy H Lakey, David A. Fulton

Corresponding authors: [alexandra.solovyova@newcastle.ac.uk](mailto:alexandra.solovyova@newcastle.ac.uk); [david.fulton@newcastle.ac.uk](mailto:david.fulton@newcastle.ac.uk)

Simulation of a disordered Caf1 monomer scattering curve.

When thermally denatured at 90°C, Caf1 is believed to be a disordered monomer until it joins the repolymerizing oligomeric chain (Zavialov et al. 2003). To evaluate a possible contribution of disordered Caf1 monomers to the total scattering intensity during the time course, we generated a scattering curve produced by a Gaussian chain of similar size. The scattering intensity at zero-angle (I(0)) was estimated from the atomic composition of the Caf1 monomer using the SoMo SAXS program for the sample concentration of 5 mg/mL, and the radius of gyration for the chain was calculated as , where y=L/b (Perez et al. 2001). The chain contour length, L, can be expressed as L= n×a×f, where n denotes the number of amino acid residues in the polypeptide (in the case of Caf1 it is 149 amino acid residues), a=3.78 Å is the characteristic dimension of one residue and f is the geometrical factor, equal to 0.95, which takes into account the constraints of polypeptide chain (Perez et al. 2001) The statistical length, b, was assumed to be 20Å (Rowe and López Piñeiro 1990), which is characteristic for a disordered polypeptide chain. Thus, the radius of gyration for a disordered protein monomer was calculated to be 41.5 Å. The hypothetical scattering curve was calculated in SasView (<https://www.sasview.org/>) using a Gaussian coil model (Debye 1947). To support the application of this model, three scattering curves measured for disordered proteins (SASBDB (Valentini et al. 2014) entries SASDD92, SASDDF6 and SASDEX4) were fitted to the Gaussian coil model (Fig. S1a). The model gave a reasonable agreement with the following reported values of radii of gyration: SASDEX4 entry R_g_^Gauss coil^= 26.55 Å, while the reported value of R_g_ was 24 Å; for SASDDF6 entry R_g_^Gauss coil^ =37.97 Å while the reported value R_g_ was 33 Å; and for SASDD92 entry R_g_^Gauss coil^ = 73.07 Å, while the reported value of R_g_ was 67 Å To ensure that the generated hypothetical scattering curve would have the correct mass, the calculated curve was analysed in ScÅtter (https://www.bioisis.net/tutorials/9) to estimate the molecular weight of the hypothetical scatter. The obtained value for the mass was 16 kDa, in reasonable agreement with the molecular weight of Caf1 monomer (15.63 kDa).





**b**

**a**

Fig.S1. The publically available scattering curves of disordered proteins (SASBDB accession codes SASDD92, SASDDF6 and SASDEX4) fitted to the Gaussian coil model (a) and hypothetical scattering curve generated as the Gaussian coil with R_g_=41 Å and I(0)_Caf1 monomer_ = 0.061 cm^-1^ (which corresponds to the sample concentration of 5 mg/mL) superimposed with the experimental scattering curves of Caf1 collected at the four initial time points of the time course (7.5, 9, 10.5 and 12 min of the repolymerisation process, the sample concentration was 5 mg/mL)(b).


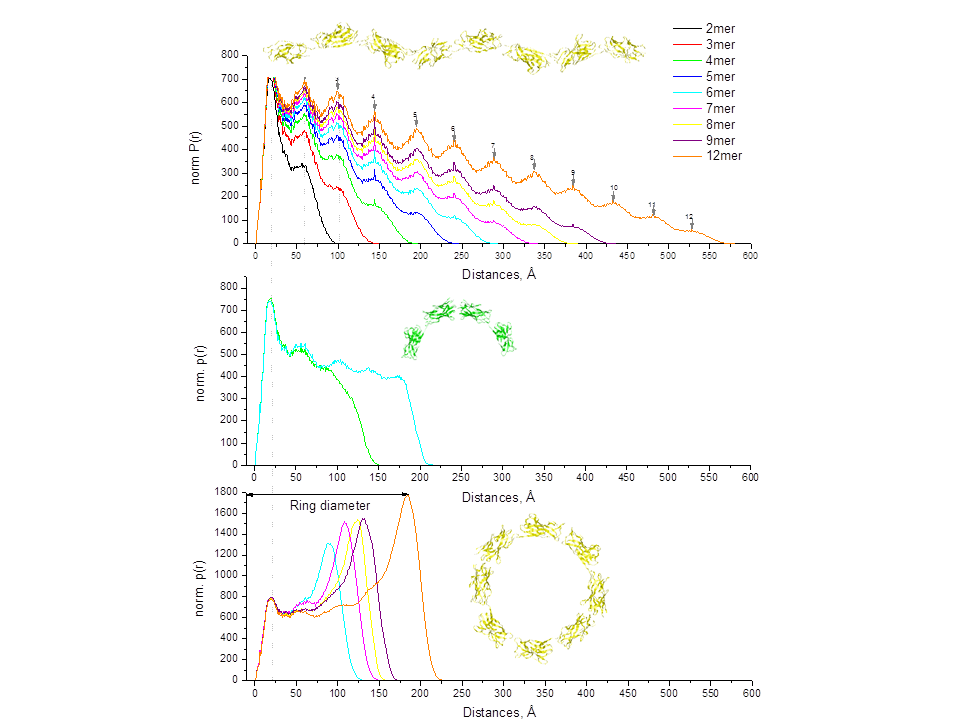


Figure S2. Calculated distance distribution function P(r) for different Caf1 oligomers using the SoMo SAS module. Oligomeric structures were modelled basing on Caf1 dimer atomic coordinates (the PDB accession code 1P5U). We show typical structures and the corresponding calculated P(r) distribution using the same colour code in all three panels.

| 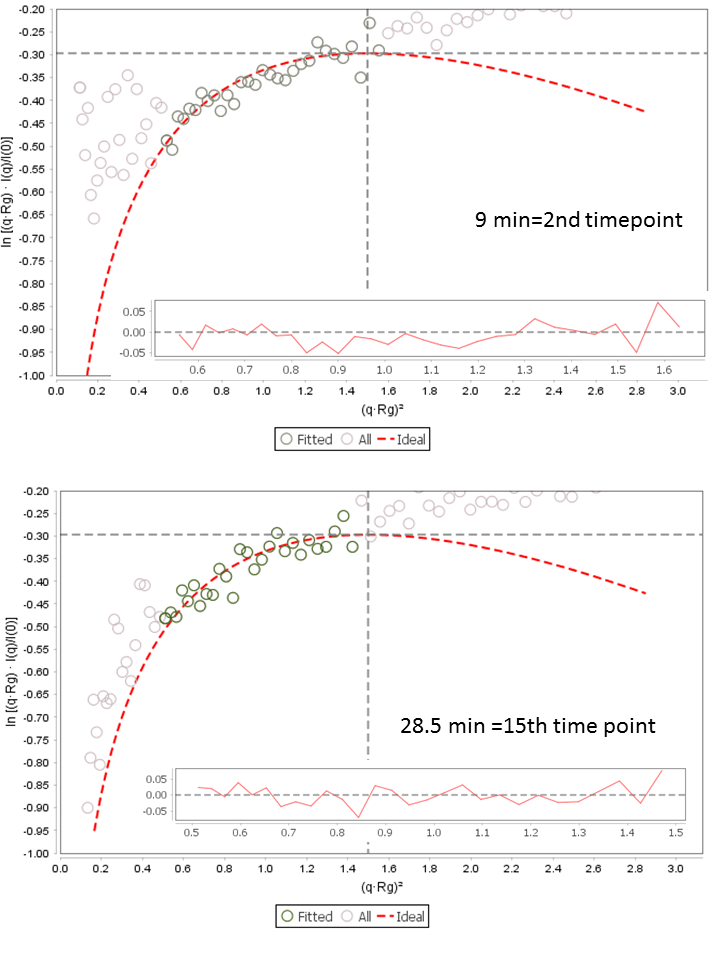 | 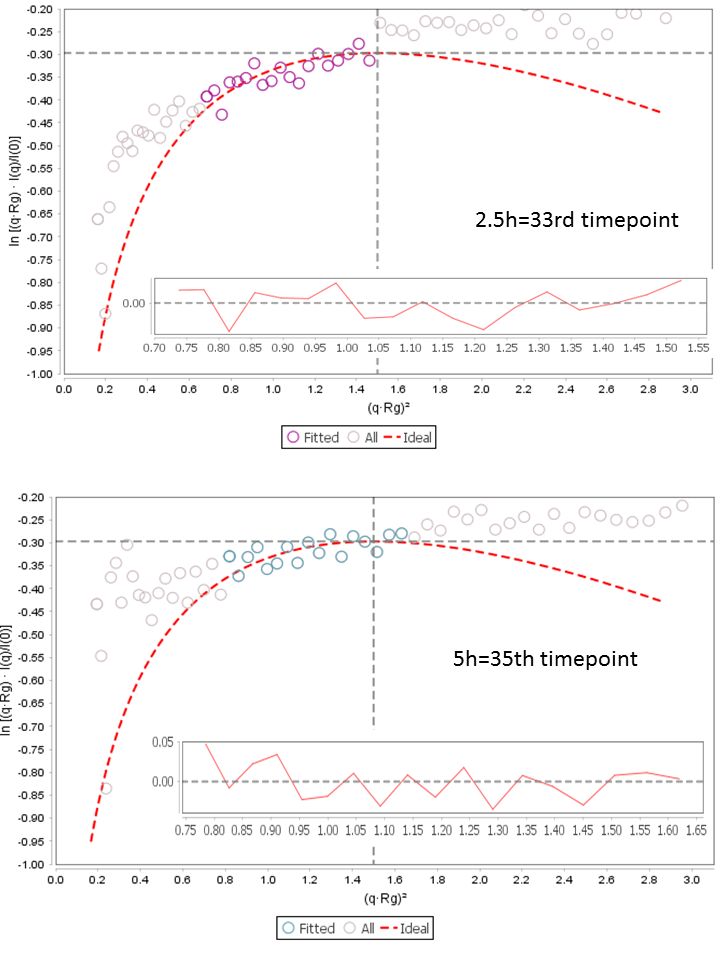 |
| --- | --- |

Figure S3. Illustrative examples of the Guinier peak analysis applied to validate the selected linear region for the Guinier approximation. The examples were taken from the scattering curves of 5mg/mL concentration set

|   a |   b |
| --- | --- |
|   c | |

Figure S4. The evaluation of the Caf1 cross-section radius of gyration from the SAXS data

a: the time course at 5 mg/mL,

b: the native Caf1 at 5 mg/mL, four replicates

c: the values of <R_c_>_z_ obtained during the first hour of the Caf1 repolymerization time course. In the insert (panel c) the data are shown for the whole time course together with native Caf1. Straight lines indicate the mean values of <R_c_>_z_ in the same colour code as the data points.





Figure S5. The dimensionless Kratky plot illustrates the shape evolution of repolymerising Caf1 during the time course (the 5 mg/mL concentration series). The curves collected at the beginning of the time course (7.5 and 13.5 min) show some elevation above zero on the qR_g_ –axis, which is indicative of the presence of some disordered structures. The cumulative smooth shape of the dimensionless Kratky plot suggests the flexible nature of Caf1 repolymerised chains (Bernado 2010). As the time course progresses, the maximum of shallow peak drifts towards higher values on qR_g_ –axis, implying the elongation of the polymeric chains.





Figure S6. <$R$_g_>z values determined from the Guinier approximation and the distance distribution function (P(r)) for all time course measurements and three sample concentrations.





Figure S7. The residuals’ plots showing the goodness of the form-factor model fit at 8 illustrative time points for repolymerising Caf1 at 5 mg/mL. The scattering curves were fitted to four form factor models: rigid cylinder (green line), flexible cylinder (black line), fractal (blue line) and polymer with excluded volume (red line).





Figure S8. <$R$_g_>z values obtained in reciprocal space (i.e from the Guinier approximation) and real space (from P(r) distribution) compared with the <$R$_g_>z values obtained from the form-factor model. The color scheme for the datapoints is the same as for the form-factor model data fit residues (Fig. S7).







Figure S9. Fast cooling (on ice) of monomerised Caf1 results in the formation of polydisperse species in solution at the early stages of repolymerisation (top panel), while slow cooling (at room temperature) produces virtually monodisperse solution (bottom panel).





b

a

Figure S10. <R_g_>_z_ (a) and <N>_w_ (b) time dependencies evolving as Caf1 repolymerisation was triggered on ice and at the room temperature. The sample concentration in both cases was 5 mg/mL. The samples, which have been placed on ice after thermal monomerization, repolymerise more quickly. The significantly larger <R_g_>_z_ values determined in Guinier approximation demonstrate the polydispersity in the fast growing samples (repolymerisation was triggered by post-denaturation cooling on ice), compared to the fit of the polymer with excluded volume model with the contribution of polydispersity omitted. In slow growing samples (where repolymerisation was initiated at room temperature) both models give similar values of <R_g_>_z_.


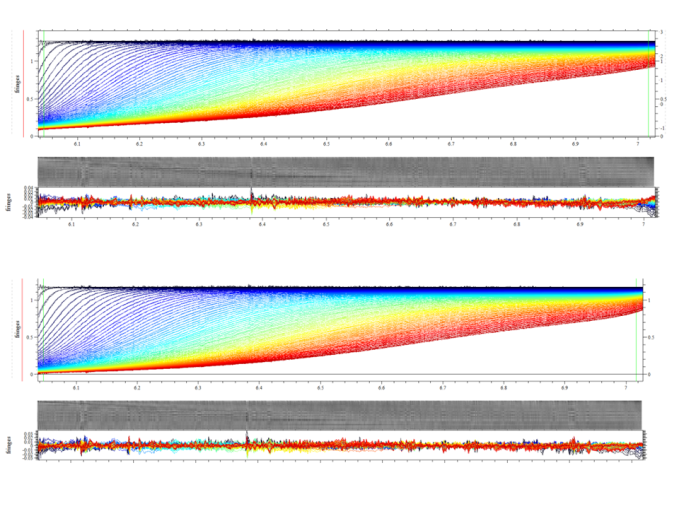


a

b


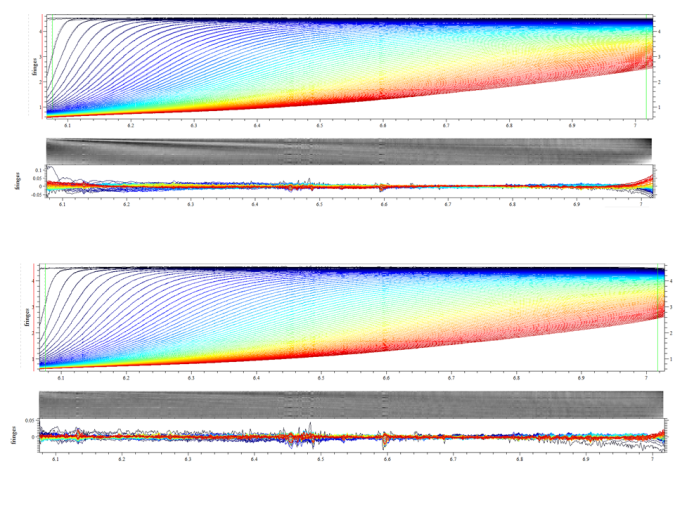


c

d

Figure S11. An example of the sedimentation velocity boundaries collected at 20,000 rpm (a): 5 mg/mL repolymerising sample, the examined sample concentration was 0.38 mg/mL 3 days post-denaturation, analysed as 1D c(*s*) distribution, rmsd=0.0056 (b) the same dataset analysed with a 2D model c(*s, f/f_0_*) rmsd = 0.0045; (c) 5 mg/mL repolymerising sample, the examined sample concentration was 1 mg/mL and run 60 days post-denaturation analysed as 1D c(*s*) rmsd = 0.009; (d) the same set of boundaries analyzed as2D c(*s, f/f0*) size distribution model rmsd = 0.0045


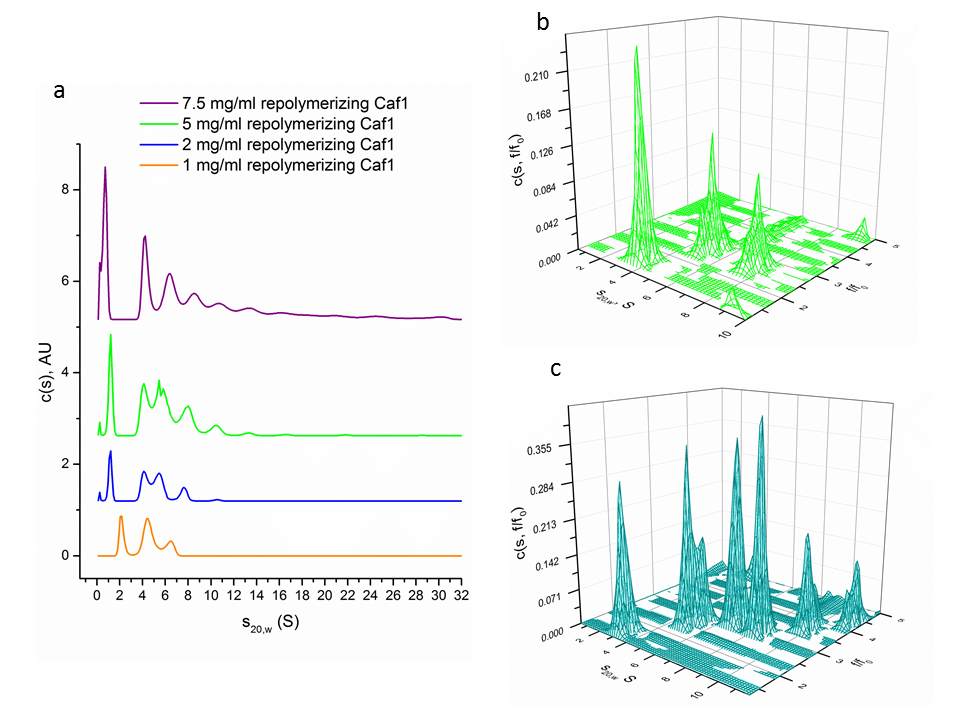


Figure S12. The results of sedimentation velocity experimentrepresented in form of c(s) (1D) (a) and c(*s, f/f0*) (2D) (b) and (c).

(a) various re-polymerizing sample concentrations 3 days post denaturation.

(b) and (c) 5 mg/mL repolymerising Caf1s sample run 3 and 60 days post denaturation respectively

References

Bernado P. 2010. Effect of interdomain dynamics on the structure determination of modular proteins by small-angle scattering. European Biophysics Journal 39:769-780.

Debye P. 1947. Molecular-weight determination by light scattering. J Phys Colloid Chem 51:18-32.

Perez J, Vachette P, Russo D, Desmadril M, Durand D. 2001. Heat-induced unfolding of neocarzinostatin, a small all-beta protein investigated by small-angle X-ray scattering. J.Mol.Biol. 308 721-743.

Rowe G, López Piñeiro A. 1990. Influence of the solvent on the conformational-dependent properties of random-coil polypeptides. I. The mean-square of the end-to-end distance and of the dipole moment. Biophys Chem 36:57-64.

Valentini E, Kikhney AG, Previtali G, Jeffries CM, Svergun DI. 2014. SASBDB, a repository for biological small-angle scattering data. Nucleic Acids Research 43:D357-D363.
